# Unique genetic signatures in HIV-1 subtype A1 and A1D recombinant envelope glycoprotein distinguish contemporary transmitted/founder viruses from historical strains in East Africa

**DOI:** 10.3389/fmicb.2025.1632581

**Published:** 2025-08-04

**Authors:** Frank Kato, Anne Kapaata, Ronald Galiwango, Angella Nakyanzi, Christian Ndekezi, Fortunate Natwijuka, Denis Omara, Andrew Ekii Obuku, Brian Foley, Pontiano Kaleebu, Eunice Nduati, Sheila Nina Balinda

**Affiliations:** ^1^MRC/UVRI & LSHTM Uganda Research Unit, Department of Viral Pathogens, Entebbe, Uganda; ^2^Department of Immunology and Molecular Biology, College of Health Sciences, Makerere University, Kampala, Uganda; ^3^The African Centre of Excellence in Bioinformatics and Data Intensive Sciences, Kampala, Uganda; ^4^The Infectious Diseases Institute, Makerere University, Kampala, Uganda; ^5^Uganda Virus Research Institute, Entebbe, Uganda; ^6^Theoretical Biology and Biophysics, Los Alamos National Laboratory, Los Alamos, NM, United States; ^7^KEMRI Wellcome Trust Research Programme, Kilifi, Kenya

**Keywords:** HIV-1, envelope, subtype A1, A1D recombinants, signature, transmitted/founder, genetic

## Abstract

**Introduction:**

The envelope glycoprotein (Env) of HIV-1 Transmitted/Founder (T/F) viruses in subtypes B and C carries distinct genetic signatures that enhance transmission fitness, augment infectivity and immune evasion. However, there is limited data on such signatures in T/F subtypes A1, D and A1D recombinants that predominate East Africa’s HIV epidemic.

**Methods:**

We used phylogenetically corrected approaches to detect distinct genetic signatures by comparing 44 contemporary HIV-1 T/F Envs with 229 historical Envs of the same subtype in East Africa.

**Results and Discussion:**

Subtype analysis based on the full-length Env gene of contemporary T/F viruses revealed a high proportion of subtype A1, followed by A1D recombinants, and fewer subtype D. Signature analysis revealed that the contemporary subtype A1 T/Fs were more likely to select distinct amino acids, including M22 in the signal peptide, R82 in gp120, A172 in the V2 loop, E230 in the glycosite 230, K275 in the D loop, Y317 in the V3 loop, K476 and N477 in the CD4 contact site, when compared with the historical Envs (q-value < 0.2). Conversely, the contemporary subtype A1 T/F Envs were less likely to carry the amino acids Q432 in the CD4 contact site, and the L784 signature within the LLP-2 (q-value < 0.2). The A1D recombinant T/Fs were more likely to select the D620 in the C-helix, but under selected the L34 in gp120, P299 in the V3 loop and Y643 in the Heptad repeat-2, compared to the historical Envs (q-value < 0.2). The distinct signature sites reported in this study may contribute to the successful establishment of acute infection as well as the persistence of long-term infection. Therefore, effective therapeutics and vaccines may target these distinct amino acid signatures especially for the East African region as it may be necessary to employ subtype-specific vaccines according to the subtype distribution.

## 1 Introduction

HIV-1 remains a major health concern, particularly in East and Southern Africa. Vaccine development has been hampered by viral genetic diversity and immune escape. HIV group M, responsible for the global pandemic, is divided into 10 subtypes (A-D, F-H, J, K, and L), six A subtypes (A1-A6), F subtypes (F1-F2), circulating recombinant forms (CRFs) and unique recombinant forms (URFs) (Désiré et al., 2018; Reis et al., 2019; Robertson et al., 2000; Yamaguchi et al., 2020). In the East African region, subtypes A1, D, and a recent increase in A1D recombinants drive the pandemic (Balinda et al., 2022; Bbosa et al., 2019; Grant et al., 2020; Adhiambo et al., 2021; Kemal et al., 2013; Yang et al., 2004). The increasing prevalence of A1D recombinants may reflect selective pressures against pure subtype D, which exhibits lower transmissibility (Kiwanuka et al., 2009), faster rates of CD4 T cell loss (Kaleebu et al., 2002) and faster disease progression (Kapaata et al., 2021; Ssemwanga et al., 2013).

The most diverse gene of HIV-1 is the Env, comprising of gp41 and gp120 which are associated with viral transmission (Herbeck et al., 2006) and host cell tropism (Yang et al., 2004). Although the Env is the sole target for neutralizing antibodies (Wibmer et al., 2015), its immense diversity regulates the functional properties of the virus and aids in rapid evolution, leading to the establishment of a viral reservoir that hinders cure and vaccine development (Ndung’u et al., 2019; Van Regenmortel, 2017; Zhou et al., 2021). The Env (gp160) encoded by the viral genome harbors the transmembrane domain and interacts with the cell surface-associated receptor (CD4) and coreceptors (CCR5 and CXCR4) by one of its non-covalently associated gp120 subunit spikes linked to gp41 (Checkley et al., 2011).

Among the broad repertoire of viral variants that circulate in an infected individual, the T/F virus that successfully establishes productive infection in a recipient is of utmost importance for prevention strategies. Understanding key genetic features of contemporary T/F viruses provides insights into mechanisms underlying transmission, which is important for both vaccine design and therapeutic interventions. Unique genetic signatures have been identified among subtype B T/F Env sequences, for example a Histidine signature in position 12 in the signal peptide (SP) and loss of an N-linked glycosylation site at positions 413–415 were associated with high Env expression levels in acute infection. This indicates that immune evasion patterns that recur in many individuals during chronic infection when antibodies are present can be selected against when the infection is being established (Gnanakaran et al., 2011). Also, an Isoleucine at position 841 instead of arginine in gp41 CT (LLP-1) was enriched in subtype B Env (Kafando et al., 2019). A K6I mutation located at the signal peptide region was found more likely among chronic viruses than the T/F viruses among subtype B Env (Kafando et al., 2019). The RV144 vaccine signature sites; lysine at position 169 in V2 and Isoleucine at position 307 in V3 loops occurred less in subtype C T/F Env sequences compared to chronic viruses (Rademeyer et al., 2016). The above studies emphasize that HIV-1 T/F viruses possess inherent properties for establishment in a new host. However, there is limited data on the unique genetic signatures in the Env for HIV-1 T/F subtypes A1, D, and A1D recombinants circulating in the East African region.

To address this gap, we utilized Single genome amplification (SGA), Sanger sequencing, Entropy tool, GenSig tool, and analyze align tool to detect the distinct genetic signatures in the Env gene of contemporary HIV-1 T/F viruses from the East African region. These were compared to historical viruses of the same subtype. Additionally, the HIV genome browser was deployed for associating the detected genetic signatures with Env protein structural characteristics to predict their sensitivity or resistance to broadly neutralizing antibodies (bnAbs).

## 2 Materials and methods

### 2.1 Study design

This was a cross-sectional study involving laboratory data generated for the contemporary HIV-1 T/F and historical viral sequences downloaded from the Los Alamos National Laboratory HIV sequence database (LANL HIV sequence db). To generate the full-length T/F Env, we used samples from the International AIDS Vaccine Initiative’s (IAVI) Virus Surveillance study working with recent infection cohorts, i.e., Protocol N in Kenya, Good Health for Women Project (GHWP) and KILGORIS at the MRC/UVRI and LSHTM Uganda Research Unit. The samples were collected between 2015 and 2021. Briefly, GHWP was a prospective cohort of women at risk who are involved in commercial sex in Uganda (Mayanja et al., 2020; Vandepitte et al., 2013). KILIGORIS cohort was a pilot study to evaluate the possibility of identifying, enrolling and following up a high-risk cohort of acute HIV infection in Uganda. Protocol N was an observation study to determine the immune and viral characteristics during acute infection in Kenya. Protocol N participants were identified from high-risk populations, e.g., Protocol B, where, despite HIV prevention care and counselling, a small proportion of HIV incidence cases occurred (Price et al., 2020). The cohort characteristics of these early infection cohorts are shown in Supplementary Table S2. We purposively selected acutely infected samples from participants who had seroconverted to HIV-1, screened for early HIV infection and those between Fiebig stage 1 to V. These acute samples were included in the present study if sufficient plasma was sufficient for Single Genome Amplification analysis. The contemporary T/F Envs from early infection cohorts were supplemented with publicly available and similar T/F Env sequences from IAVI’s protocol C in Rwanda. These additional T/F Envs from Rwanda were retrieved from the CATNAP webserver http://hiv.lanl.gov/catnap (Umviligihozo et al., 2021; Yoon et al., 2015) and Super Filtered (SFL) web alignments https://www.hiv.lanl.gov/content/sequence/NEWALIGN/align.html#filter at LANL HIV db. Also, the historical full length Env sequences from Uganda, Kenya, Tanzania and Rwanda were downloaded from the CATNAP webserver and SFL web alignments at LANL HIV db.

### 2.2 Ethical consideration

Ethics approvals were obtained for the GHWP from Uganda Virus Research Institute-Research and Ethics Committee (UVRI-REC) (GC 127). Protocol N cohort was approved by the KEMRI Scientific and Ethics Review Unit (SERU) (KEMRI/RES/7/3/1). Kiligoris cohort was approved by UVRI-REC (GC/127/714). Also, this study was approved by the School of Biomedical Sciences-Research and Ethics Committee (SBS-REC) (Ref: SBS-2023-38) at Makerere University. The permission to use the archived plasma samples (from GHWP, Protocol N and Kiligoris cohorts) was sought from the MRC/UVRI and LSHTM Uganda Research Unit.

### 2.3 Sample size

In this study, full-length contemporary HIV-1 T/F Env sequences (*n* = 44) were compared with historical Env sequences (*n* = 229) of similar subtypes from East Africa. Contemporary T/F Env sequences were generated from the laboratory at the MRC/UVRI and LSHTM Uganda Research Unit from recent cohorts as follows: GHWP (*n* = 18), Protocol N (*n* = 11), and KILIGORIS (*n* = 7). The accession numbers of the publicly available contemporary T/F Envs from IAVI’s Protocol C (*n* = 8) and historical sequences retrieved from the LANL HIV db are available in the Supplementary Tables S3–S6. The historical Env sequences comprised of old acute (*n* = 24), chronic (*n* = 200) and viruses of unknown infection phase (*n* = 5), and spanning the years 1986 to 2006.

### 2.4 Viral RNA isolation and cDNA synthesis

The Viral RNA was extracted from 140 μL of archived EDTA plasma (from the IAVI’s protocol N, GHWP and Kiligoris cohorts) using the Qiagen viral RNA extraction kit (Qiagen Inc., Valencia, CA, United States) following the manufactures’ instructions. The recovered RNA was converted into complimentary DNA (cDNA) using SuperScript IV reverse transcriptase (Invitrogen, Ljubljana, Slovenia) as previously described (Salazar-Gonzalez et al., 2009). The cDNA templates for complete HIV-1 Env single genome amplification were synthesized with the reverse primer 1. R3B3R (Supplementary Table S1). The cDNA was used immediately to generate single genome amplicons.

### 2.5 Single genome amplification

The event of inter-subtype recombinants *in vivo* and artificial recombinants that may be generated *in vitro* because of template switching during bulk amplification of heterogeneous cDNA target sequences confound earlier findings on acute HIV-1 infection. A common strategy to tackle these challenges has been to identify participants within the acute phase of infection and using SGA, derive viral sequences from proviral DNA or plasma RNA, followed by sequencing, and phylogenetic analysis (Salazar-Gonzalez et al., 2009). Similarly, we serial diluted the cDNA and performed nested PCR amplification with HIV-1 specific primers (Supplementary Table S1) as previously described (Salazar-Gonzalez et al., 2009). All products derived from cDNA dilutions and PCR amplifications yielding <30% positive wells and amplicon length (±2.6-kb) were subjected to Sanger sequencing.

### 2.6 Sanger sequencing

To confirm amplification from single cDNA templates and avoid *in vitro* PCR artefacts, 5–10 SGA derived amplicons per participant were sequenced using BigDye Terminator v3.1 chemistry (Applied Biosystems, Foster City, CA), and an Applied Biosystems 3500xl Genetic analyzer (Thermo Fisher Scientific, Foster City, CA, United States).

### 2.7 HIV-1T/F identification from recent infection cohorts

The raw sequence files were acquired from the genetic analyzer, base-called and *de novo* assembled using the Sequencher program (v5.4.6; Gene Codes, Ann Arbor, MI) (Salazar-Gonzalez et al., 2008). The assembled full-length Env sequences were aligned using MAFFT package v7.505 (Katoh et al., 2002). Maximum likelihood tree construction for the aligned SGA Env sequences was done using IQ-TREE v2.0.3 (Nguyen et al., 2015; Trifinopoulos et al., 2016), with free rate of evolution, GTR model, and 1,000 bootstrap replicates, including multiple sequences from each participant to rule out contamination issues. The tree file (Newick format) was visualized using the Figtree package v1.4.4 (Rambaut, 2010). To identify and enumerate contemporary HIV-1 T/F variants, we used maximum likelihood tree reconstruction for within participant-specific phylogenetic clustering and the LANL HIV sequence visualization tool https://www.hiv.lanl.gov/content/sequence/HIGHLIGHT/highlighter_top.html to generate highlighter plots and determine recombinant mosaic structures between cognate major and minor T/F variants (Balinda et al., 2022; Keele et al., 2008; Macharia et al., 2020).

### 2.8 Retrieval of historical Env sequences

The historical dataset (*n* = 229) comprised of old acute, old chronics and old Env sequences from HIV-1 viruses of unknown infection status. These were retrieved from the LANL HIV db using filters: HIV-1, subtype (either A1, D or A1D recombinants), Env CDS, patient health, days from seroconversion, Fiebig stage, days from infection, infection year, Sub-Saharan Africa, including sequences with less than 0.5 Percent non-ACGT to rule out problematic sequences. Next, we applied the one sequence per patient filter to rule out bias introduced by analyzing multiple sequences from one patient. Sequences were retrieved from LANL HIV-1 super filtered (SFL) alignments using filters: HIV-1/SIVcpz, DNA, and All M group with CRFS. In addition, sequences were retrieved from CATNAP Env alignment at LANL HIV db. This was followed by an extensive literature search and exclusion of sequences out of the sampling year range (1986–2006), non-subtype A1, non-subtype D, non-A1D recombinants and sequences sampled outside the East African region.

### 2.9 Retrieval of contemporary T/F Env sequences

The contemporary HIV-1 TF Env sequences (*n* = 8) from acute infection were retrieved using the procedure in 2.8, followed by an extensive literature search and exclusion of sequences outside the sampling year range (2015–2021), chronic and sequences of unknown infection status, non-subtype A1, non-subtype D, and non-A1D recombinants and those sampled outside the East African region.

### 2.10 HIV-1 subtype analysis

The full-length HIV-1 T/F Env sequences were subtyped using the Recombinant identification Program (RIP, window size = 400) (Siepel et al., 1995) and jumping profile Hidden Markov Model (jpHMM) (Schultz et al., 2009).

### 2.11 Quality control

The contemporary T/F and historical Envs from East Africa were codon aligned using Gene Cutter https://www.hiv.lanl.gov/content/sequence/GENE_CUTTER/cutter.html. The codon-aligned sequence alignment was fed into ElimDupes tool https://www.hiv.lanl.gov/content/sequence/elimdupesv2/elimdupes.html to eliminate 100% identical sequences with consideration of sub-sequences as duplicates. The unique sequences from the ElimDupes tool were subjected to the quality control tool at LANL HIV db https://www.hiv.lanl.gov/content/sequence/QC/index.html to exclude sequences with stop codons and non-ACGT characters within the Env CDS. Maximum likelihood trees were reconstructed in IQ-TREE v2.0.3 using free rate of evolution, 1,000 bootstrap replicates and the GTR model to eliminate the sequence far away from the root in cases of homogeneous or clustering sequences (Trifinopoulos et al., 2016). The quality-controlled sequences were then used for entropy and unique genetic signature analysis. The detected signatures were associated with Env structural characteristics such as sensitivity or resistance to bnAbs using the HIV genome browser at LANL HIV db. These analyses were stratified by each subtype (A1, and A1D recombinants).

### 2.12 Entropy analysis of contemporary HIV-1T/F compared to historical Env sequences

For each alignment position, the Entropy-two tool https://www.hiv.lanl.gov/content/sequence/ENTROPY/entropy.html was deployed to compare the variability in contemporary T/F Env subtype A1 (query) relative to historical subtype A1 Env (background). To guide against type 1 errors, the false discovery rate (q-value) for *p*-values (from Entropy tool) was determined using the source code https://github.com/nfusi/qvalue (Storey and Tibshirani, 2003). All significant entropy sites (q-value < 0.2) were visualized using Analyze align tool https://www.hiv.lanl.gov/content/sequence/ANALYZEALIGN/analyze_align.html. The entropy analysis procedure was repeated for A1D recombinants.

### 2.13 Genetic signature analysis of contemporary HIV-1T/F compared to historical Env sequences

Using the GenSig tool at LANL HIV sequence db, we performed a phylogenetically corrected analysis to detect unique genetic signatures in the codon aligned DNA alignment of contemporary T/F subtype A1 and historical subtype1 A1 Envs (Bhattacharya et al., 2007; Bricault et al., 2019). The GenSig searched for statistically significant signatures with a site depth of 1 which tests for the association between the contemporary T/F and historical Envs, and each amino acid in all independent sites in the alignment (Bhattacharya et al., 2007; Bricault et al., 2019). The Gensig tool provides *p*-values for Fisher’s exact test and corresponding q-values in the signature output to minimize false positives due to lineage effects (Bhattacharya et al., 2007; Bricault et al., 2019). The absolute counts, frequency by position and weblogs for the significant genetic signatures (q-value < 0.2) were generated using Analyze align tool. The genetic signature analysis procedure was repeated for the A1D recombinants.

### 2.14 Exploration of the association between the unique genetic signatures and HIV-1T/F Env characteristics

The LANL HIV db genome browser tool was used to investigate the association between the significant genetic signatures and the HIV-1 T/F Env subtype structural characteristics (Skinner et al., 2009; Skinner and Holmes, 2010). Briefly, the Env option on the HIV genome browser was selected, dragged the tracks to the top left section and entered the HXB2 numbering position of the significant signature site in the search box. Also, the unique genetic signatures were associated with available bnAb data in the “HIV-1 Neutralizing Antibody signatures and applications to epitope targeted vaccine study” (Bricault et al., 2019).

## 3 Results

### 3.1 Identification of contemporary HIV-1T/F and historical Env sequences

**Figure 1:**
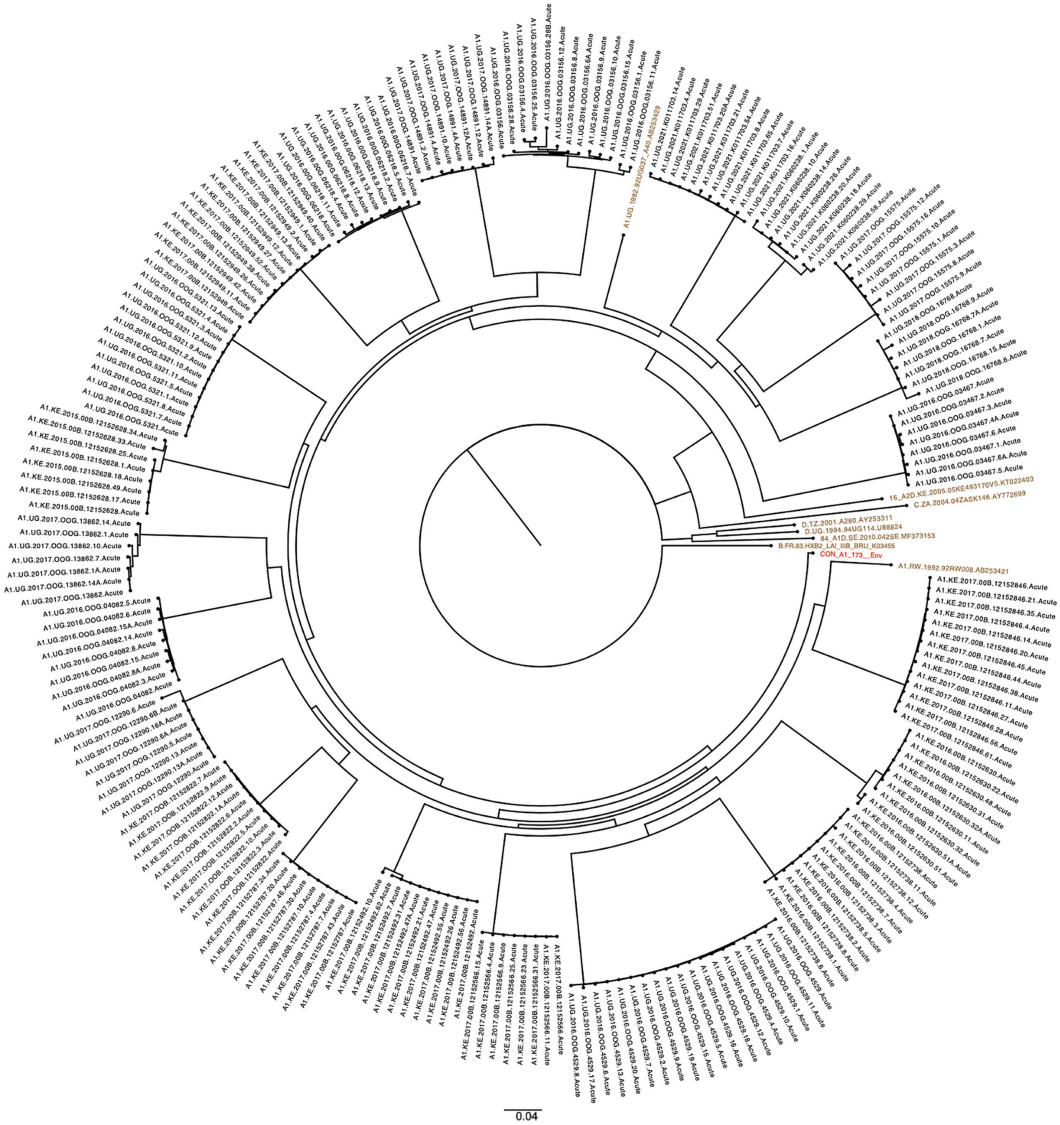
SGA-derived subtype A1 Env sequences (*n* = 221) from participants (*n* = 22) clustered into distinct lineages indicating Single transmission events. HIV-1 reference sequences (brown) and group M subtype A1 consensus sequence (red) from the LANL HIV db. The scale bar represents genetic distance.

**Figure 2:**
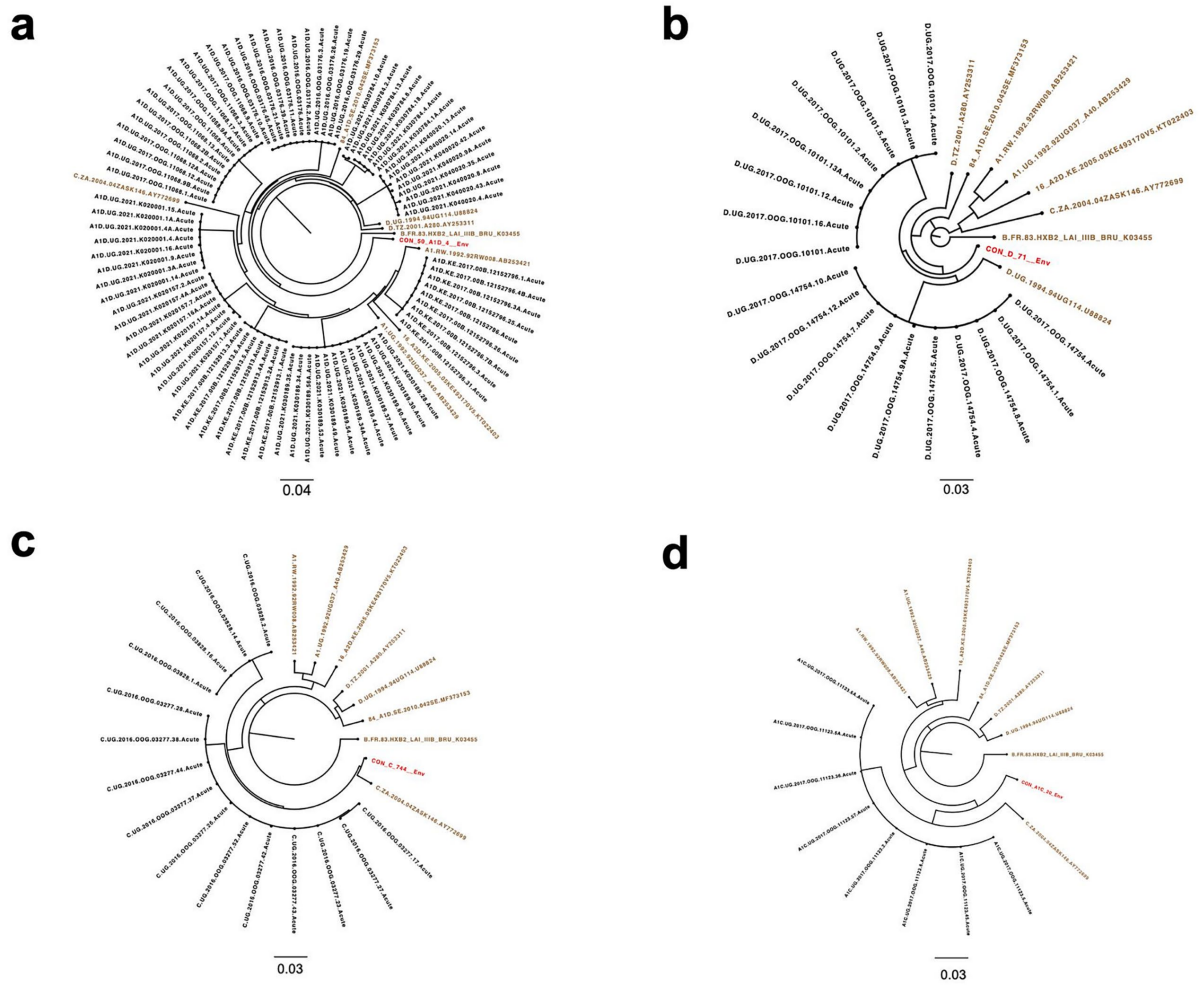
Maximum likelihood phylogenetic trees of SGA-derived Env sequences indicating single transmission events in each participant. **(a)** Subtype A1D recombinant Env sequences (*n* = 82) from participants (*n* = 9). **(b)** Subtype D Env sequences (*n* = 18) from subjects (*n* = 2). **(c)** Subtype C SGA sequences (*n* = 15) from participants (*n* = 2). **(d)** Subtype A1C Env sequences (*n* = 8) from participant (*n* = 1). HIV-1 reference sequences (brown) and group M subtypes D, C, A1D and A1C recombinant consensus sequences (red) from the LANL HIV db. The scale bar represents genetic distance.

A total of 344 SGA-derived viral sequences from 36 acutely infected individuals (GHWP, Protocol N and KILIGORIS Cohorts) were phylogenetically analyzed to infer 36 contemporary T/F Env sequences. Across the acute cohort, the mean, median and range numbers of SGA-derived sequences per participant are 9.56, 9 and 15, respectively, as shown in the Supplementary Table S2. Maximum likelihood phylogenetic trees formed subject-specific lineages that conformed to a single transmission event Figures 1, 2a–d. The identified T/F Env sequences from early infection cohorts were supplemented with available T/F Envs (*n* = 8) from LANL HIV sequence db, resulting in 44 T/F Env sequences for this study. These were compared with available historical Envs (*n* = 229) from LANL HIV db.

**Figure 3:**
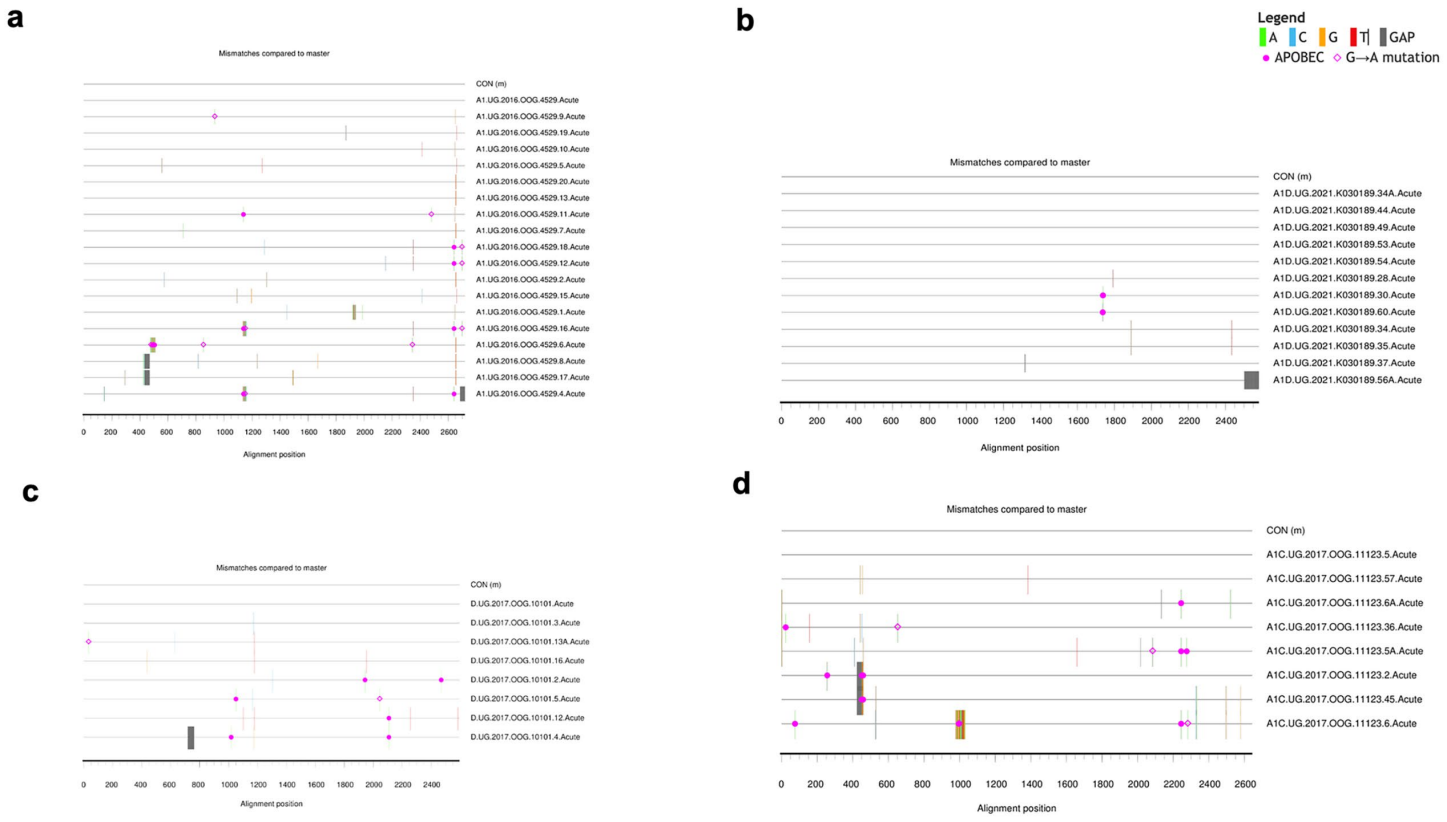
Highlighter plots. Representative subjects with single virus transmission **(a–d)**. Tic marks represent nucleotide substitutions as compared to the top-most master (consensus) or T/F sequence in each highlighter plot.

Additionally, the highlighter plot analysis showed those individuals infected by a single virus (Figures 3a–d). In all single transmission events, mismatches compared to the master (consensus) in each amplicon were randomly distributed across the complete HIV-1 Env genome. The sequence that corresponded to the consensus sequence was inferred to be the T/F sequence.

### 3.2 HIV-1 Env subtype A1 was more predominant than A1D recombinant and subtype D

**Figure 4:**
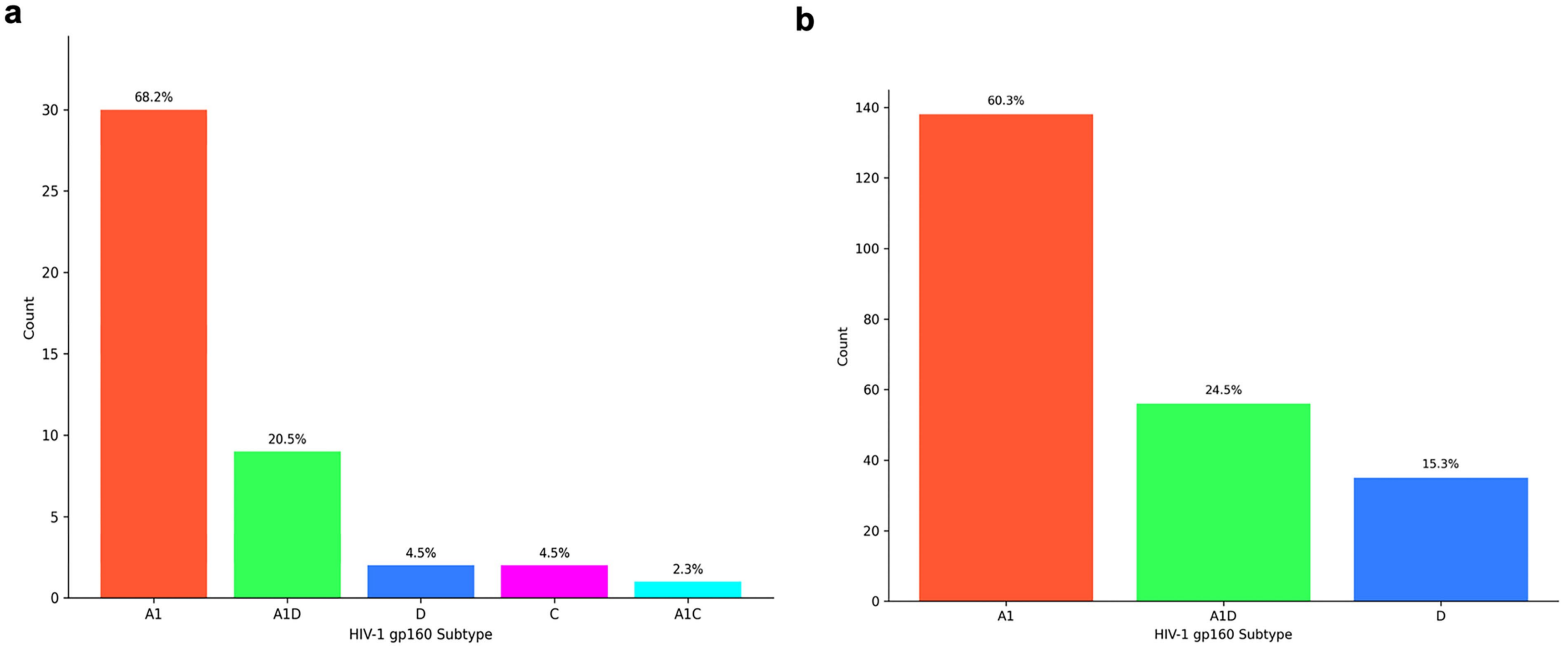
Distribution of HIV-1 envelope subtypes in East Africa according to RIP and jpHMM tools. The x-axis represents the full-length Env (gp160) subtype while the y-axis represents the number of sequences **(a)** HIV-1 subtype distribution of contemporary T/F Envs from 2015 to 2021. **(b)** HIV-1 subtype distribution of historical Envs from 1986 to 2006.

The contemporary HIV-1 T/F Subtype A1 (68.2%, 30/44) was the most predominant, followed by A1D recombinants (20.5%, 9/44), subtype D (4.5%, 2/44), subtype C (4.5%, 2/44) and A1C recombinant (2.3%, 1/44) (Figure 4a). Similarly, for the historical HIV-1 Env sequences, the most prevalent subtype was subtype A1(60.3%, 138/229), followed by A1D recombinants (24.5%, 56/229) and then subtype D (15.3%, 35/229) (Figure 4b).

### 3.3 Amino acid variation in contemporary T/F compared to historical HIV-1 Envs

In the case of subtype A1, the contemporary T/F Envs were more variable at HXB2 alignment positions (22, 82, 172, 230, 275, 317, 432, 476, 477 and 784) when compared to the historical sequences (q-value < 0.2) (Table 1). Among A1D recombinants, the T/F Envs exhibited higher diversity at HXB2 alignment positions (34, 299, and 643) when compared to the historical sequences (q-value < 0.2) (Table 1). Conversely, the A1D recombinant historical Envs were more variable at position 620 when compared to the contemporary A1D recombinant T/F (q-value < 0.2). The small sample size of subtype D sequences limited the statistical power to compare the variation of amino acids between contemporary T/F and historical subtype D Env sequences.

**Table 1:** Entropy analysis of amino acids among HIV-1 Env sequences between contemporary T/F and historical viruses.

HXB2 alignment position	HXB2 amino acid	Alignment position	Query consensus	Randomized entropy diff> = Hdiff	Highest randomized entropy diff	1st seq in query set	Background entropy (Hb)	Query Entropy (Hq)	(Hdiff = Hb-Hq)	*P*-value	Q-value	HIV-1 Env subtype
**22**	L	28	L	0	0.448	L	0.043	0.703	−0.66	0	0	A1
**82**	Q	92	Q	4	0.396	Q	0.076	0.393	−0.317	0	0	A1
**172**	E	203	V	1	0.715	V	0.36	1.068	−0.707	0	0	A1
**230**	N	307	D	4	0.712	E	0.464	1.036	−0.572	0	0	A1
**275**	V	355	E	3	0.541	Q	0.19	0.674	−0.484	0	0	A1
**317**	F	399	F	4	0.523	F	0.086	0.531	−0.446	0	0	A1
**432**	K	534	Q	4	0.706	Q	0.431	1.05	−0.62	0	0	A1
**476**	R	591	R	4	0.396	R	0.179	0.543	−0.364	0	0	A1
**477**	D	592	D	2	0.325	D	0	0.325	−0.325	0	0	A1
**784**	L	902	L	3	0.586	L	0.161	0.673	−0.511	0	0	A1
203	Q	277	Q	15	0.325	Q	0	0.245	−0.245	0.01	0.4	A1
282	K	362	K	7	0.346	K	0	0.291	−0.291	0.01	0.4	A1
**34**	L	46	L	4	1.006	L	0.268	1.149	−0.881	0	0	A1D
**299**	P	361	P	4	0.783	P	0	0.684	−0.684	0	0	A1D
**620**	T	705	D	1	1.596	D	1.857	0.637	1.221	0	0	A1D
**643**	L	728	Y	5	0.547	Y	0	0.53	−0.53	0	0	A1D
145	G	180	–	8	1.722	–	2.204	0.849	1.355	0.01	0.6	A1D

Query entropy represents the variation of the contemporary T/F Env while background entropy denotes that of the historical Env for a given subtype. Hdiff is the entropy difference between background and query. The HXB2 alignment positions of the statistically significant entropy sites (q-value < 0.2) are highlighted in bold.

### 3.4 Unique genetic signatures in the HIV-1 Env associated with contemporary T/F compared to historical sequences

Table 2 presents the significant genetic signatures sites. We detected the robust Leucine signature at position of HXB2 numbering 22 (L22) in the hydrophobic core of the signal peptide (SP) domain in subtype A1 (*p*-value = 0.000613, q-value < 0.2, Fisher’s test). The robust L22 signature site was less enriched in the SP domain of T/F subtype A1 (80.00%) compared to the historical sequences (99.28%). The L22 is a robust genetic signature site because it was supported by multiple lines of evidence, i.e., both the Entropy and GenSig tools. Instead, the T/F subtype A1 sequences (6.67%) were more likely to select a Methionine (M22) signature, compared to the historical counterparts which lacked it (*p*-value = 0.031, q-value < 0.2, Fisher’s test).

**Table 2:** Unique genetic signatures associated with contemporary T/F compared to the historical HIV-1 Env sequences.

Contingency table	HXB2 number	HXB2 amino acid	Genetic signature region	Local aa	Test aa	*p*-value	T/F		BG		q-value	Odds ratio	HIV-1 Env subtype
							r1c1	r1c2	r2c1	r2c2			
t1	5	E	SP	6	I	0.000128	21	9	43	95	0.0112	5.1	A1
t2*	**22**	L	SP	28	**M**	0.031	2	28	0	138	0.166	Inf	A1
t3*	**22**	L	SP	28	**L**	0.000613	5	24	1	137	0.00739	27.6	A1
t2*	**82**	Q	gp120	92	**R**	0.00979	4	26	2	136	0.085	10.2	A1
t3*	**82**	Q	gp120	92	**Q**	0.00979	4	26	2	136	0.0455	10.2	A1
t2*	**172**	E	V2 loop	203	**A**	0.031	2	28	0	138	0.166	Inf	A1
t3*	**172**	E	V2 loop	203	**V**	0.00245	9	21	11	127	0.0184	4.88	A1
t2*	**230**	N	glycosite 230	307	**E**	0.0349	6	22	10	125	0.173	3.38	A1
t3*	**230**	N	glycosite 230	307	**D**	0.00436	9	19	13	120	0.0285	4.32	A1
t2*	**275**	V	D loop	355	**K**	0.00374	4	26	1	137	0.0537	20.5	A1
t3*	**275**	V	D loop	355	**E**	0.00486	6	24	5	133	0.0318	6.54	A1
t2*	**317**	F	V3 loop	399	**Y**	0.031	2	28	0	138	0.166	Inf	A1
t3*	**317**	F	V3 loop	399	**F**	0.00979	4	26	2	136	0.0455	10.2	A1
t3*	**432**	K	CD4 Contact Residue	534	**Q**	0.00531	8	21	10	122	0.0362	4.59	A1
t2*	**476**	R	CD4 Contact Residue	591	**K**	0.0214	5	23	6	132	0.123	4.72	A1
t3*	**476**	R	CD4 Contact Residue	591	**R**	0.0214	5	23	6	132	0.0691	4.72	A1
t2*	**477**	D	CD4 Contact Residue	592	**N**	0.00523	3	27	0	138	0.0686	Inf	A1
t3*	**477**	D	CD4 Contact Residue	592	**D**	0.00523	3	27	0	138	0.0362	Inf	A1
t3*	**784**	L	LLP-2	902	**L**	0.00918	4	25	2	134	0.0455	10.5	A1
t2*	**620**	E	c-helix	705	**D**	0.00171	3	1	1	34	0.121	66.2	A1D
t3*	**34**	L	gp120	46	**L**	0.00259	4	5	2	53	0.126	19.2	A1D
t3*	**299**	P	V3 loop	361	**P**	0.0173	2	7	0	56	0.174	Inf	A1D
t3*	**643**	H	Fusion HR-2	728	**Y**	0.0173	2	7	0	56	0.174	Inf	A1D

**HXB2 number**: HXB2 alignment position of the signature. Local **aa**: Local alignment amino acid position of the signature. **Test aa**: principal amino acid signatures tested or associated with T/F compared to historical Envs. ***p*-value**: probability calculated for the respective test. **q-value**: False discovery rate for the *p*-value. **T/F**: Transmitted/Founder. **BG**: Historical. **t**: table. r1 = row1 = feature = T/F, r2 = row 2 =!feature = historical. **t1**: results for Fisher’s exact tests without phylogenetic correction. t3^*^: results for Fisher’s exact tests with phylogenetic correction, i.e., if the amino acid being considered is “A,” t3^*^ shows the events in which the ancestral sequence had A, with a significant difference. For t3^*^, c1 = column 1 = A- >!A, c2 = column 2 = A- > A. t2^*^: results for Fisher’s tests with phylogenetic correction, i.e., if the amino acid being considered is “A,” t2^*^ displays the cases in which the ancestral sequence lacked “A.” for t2^*^, c1 = column 1 =!A- > A, c2 = column 2 =! A- >!A. An exclamation mark (!) means “not.

Another robust signature was the Glutamine detected at position 82 (Q82) in the gp120 domain of subtype A1 (*p*-value = 0.00979, q-value < 0.2, Fisher’s test). The gp120 of subtype A1 historical sequences (98.55%) are more likely to carry a robust Q82 signature compared to the T/F counterparts (86.67%). In contrast, gp120 domain of T/F subtype A1 (13.33%) was more likely to select an Arginine (R82) when compared to historical sequences (1.45%) (*p*-value = 0.00979, q-value < 0.2, Fisher’s test). Notably, a robust Valine signature detected at position 172 (V172) in the V2 loop of subtype A1 was greatly enriched in the historical sequences (92.03%), compared to the T/F counterparts (70.00%) (*p*-value = 0.00245, q-value < 0.2, Fisher’s test). Conversely, the V1 loop of T/F subtype A1(6.67%) exhibited a high frequency of the Alanine (A172) signature, which was absent in the historical sequences (*p*-value = 0.031, q-value < 0.2, Fisher’s test). Also, the robust aspartate signature detected at position 230 (D230) in the glycosite 230 of the subtype A1 was less enriched in T/F (63.33%), compared to the historical sequences (86.96%) (*p*-value = 0.00436, q-value < 0.2, Fisher’s test). Conversely, the glycosite 230 of T/F subtype A1 (23.33%) was more likely to select a Glutamic acid (E230) signature compared to the historical sequences (9.42%) (*p*-value = 0.0349, q-value < 0.2, Fisher’s test). What’s more, a robust Glutamic Acid signature at position 275 (E275) in the D loop of subtype A1 involved its lower frequency in T/F sequences (80.00%) compared to the historical counterparts (96.38%) (*p*-value = 0.00486, q-value < 0.2, Fisher’s test). Instead, the D loop of subtype A1 T/Fs (13.33%) exhibited a high frequency of the Lysine (K275) signature, which was absent in the historical sequences (*p*-value = 0.00374, q-value < 0.2, Fisher’s test). Importantly, the T/F subtype A1 sequences (86.67%) were less likely to select the robust Phenylalanine (F317) signature at position 317 compared to the historical counterparts (98.55%), in the V3 loop (*p*-value = 0.00979, q-value < 0.2, Fisher’s test). Instead, the V3 loop of T/F subtype A1 (6.67%) exhibited a higher frequency of the Tyrosine (Y317) signature, which was absent in the historical counterparts (*p*-value = 0.031, q-value < 0.2, Fisher’s test). Furthermore, the T/F subtype A1 viruses (70.00%) are less likely to select the robust Glutamine signature at position 432 (Q432) compared to the historical counterparts (88.41%), mainly in the CD4 Contact residue (*p*-value = 0.00531, q-value < 0.2, Fisher’s test). Similarly, the T/F subtype A1 (76.67%) exhibited a lower frequency of the robust Arginine signature at position 476 (R476) compared to the historical counterparts (95.65%), mainly in the CD4 Contact residue (*p*-value = 0.0214, q-value < 0.2, Fisher’s test). On the contrary, the CD4 contact residue in T/F subtype A1 (23.33%) exhibited a higher frequency of the unique K476 signature compared to the historical sequences (4.35%) (*p*-value = 0.0214, q-value < 0.2, Fisher’s test). Along the same lines, the T/F subtype A1 (90.00%) exhibited a lower frequency of the robust D477 signature compared to the historical viruses (100.00%), mainly in the CD4 Contact residue (*p*-value = 0.00523, q-value < 0.2, Fisher’s test). Conversely, the CD4 contact residue in T/F subtype A1 (10.00%) exhibited a higher frequency of the N477 signature, which was absent in the historical counterpart (*p*-value = 0.00523, q-value < 0.2, Fisher’s test). A robust Leucine signature (L784) detected in subtype A1 was greatly enriched in historical sequences (98.53%), compared to the T/F counterparts (86.21%), mainly in the LLP-2 lentiviral lytic peptide alpha helix (*p*-value = 0.00918, q-value < 0.2, Fisher’s test). The genetic signature frequencies by position and absolute counts determined by the Analyze Align tool are presented in Supplementary Table S7 for the contemporary T/F subtype A1 and Supplementary Table S8 for historical subtype A1 Envs.

**Figure 5:**
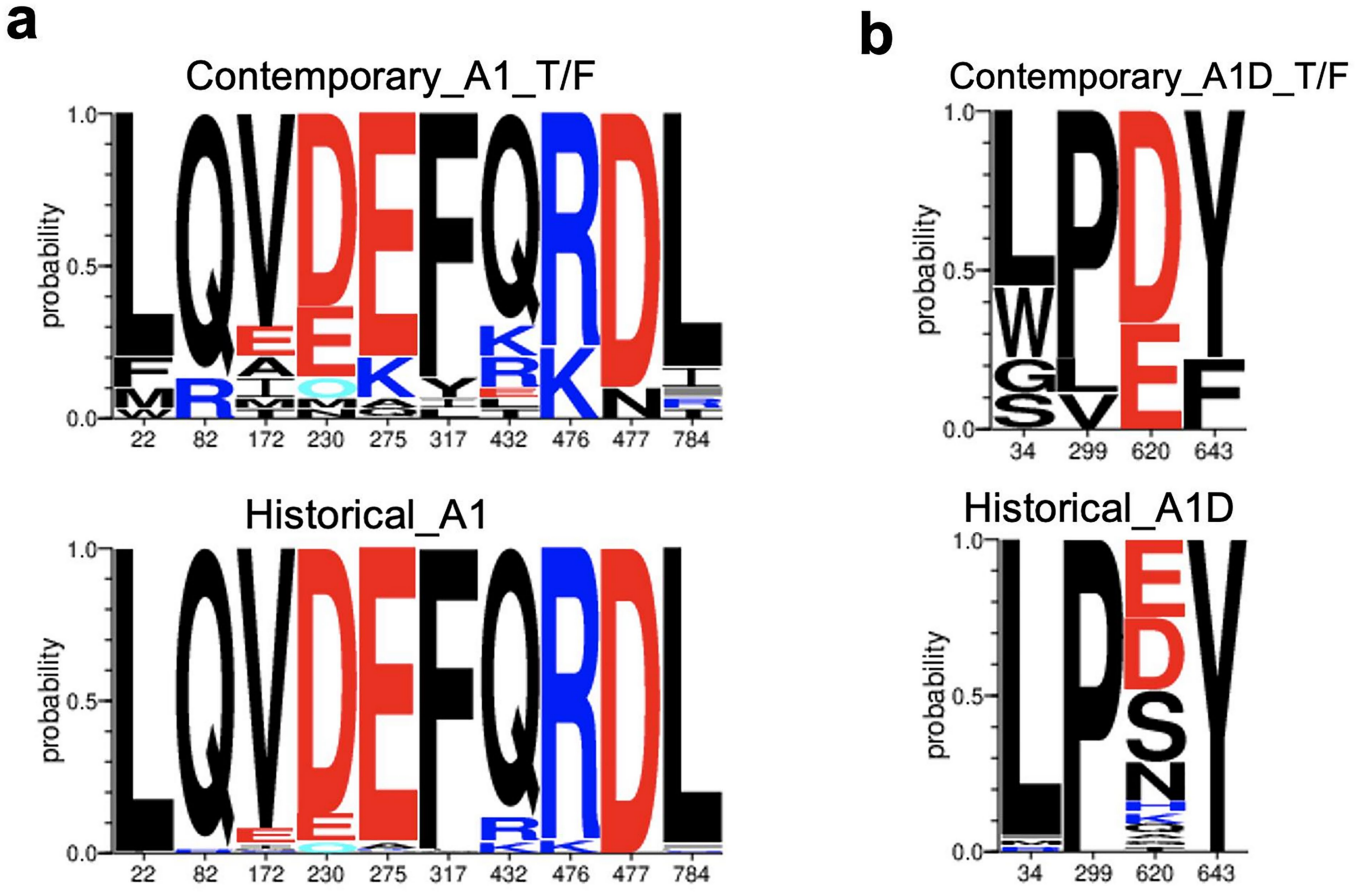
Genetic signatures identified under the HIV-1 envelope associated with contemporary T/F, compared to historical sequences. Amino acid letter probability is proportional to its relative frequency in the alignment. **(a)** Robust genetic signature sites, L22, M22, Q82, R82, V172, A172, D230, E230, E275, K275, F317, Y317, Q432, K476, R476, D477, N477, L784 associated with contemporary T/F subtype A1 (*n* = 30) compared to historical subtype A1 Envs (*n* = 138). **(b)** Robust genetic signatures, L34, P290, D620 and Y643 associated with contemporary T/F A1D recombinants (*n* = 9) compared to historical A1D recombinants (*n* = 56).

For the AID recombinants, the C-helix in the T/F (66.67%) exhibited a high frequency of the robust D620 compared to the historical counterparts (23.21%) (*p*-value = 0.00171, q-value < 0.2, Fisher’s test). Furthermore, the robust L34 signature detected in the gp120 of A1D recombinants occurred less in T/Fs (55.56%), compared to the historical counterparts (96.36%) (*p*-value = 0.00259, q-value < 0.2). The robust Proline signature detected at position 299 (P299) in A1D recombinants occurred less in the T/F sequences (77.78%) compared to the historical counterparts (100.00%), specifically in the V3 loop (*p*-value = 0.0173, q-value < 0.2). Additionally, the robust Y643 in the Fusion Heptad Repeat (HR) -2 was greatly enriched in A1D historical (100.00%) recombinants compared to the T/F counterparts (77.78%) (*p*-value = 0.0173, q-value < 0.2). The genetic signature frequencies by position and absolute counts determined by the Analyze Align tool are presented in Supplementary Table S9 for T/F A1D recombinant and Supplementary Table S10 for historical A1D recombinants. The weblogs show the robust genetic signature sites in contemporary T/F compared to the historical Env across subtypes A1, and A1D recombinants (Figure 5).

The small sample size of subtype D sequences limited our ability to detect robust or informative signatures sites in contemporary subtype D T/F Envs compared to historical counterparts.

### 3.5 Exploration of the association between the unique genetic signature sites and HIV-1 Env structural characteristics

We deployed the LANL tool Genome browser to explore the relationship between the robust and unique genetic signatures in the Env and information such as HXB2 coding sites of interest, antibody epitopes, neutralizing antibody contexts such as sensitivity and resistance. Also, the signature sites were associated with other published literature such as sensitivity or resistance to bNAbs in HIV-1 subtypes, B and C (Table 3).

**Table 3:** Genetic signature relationship to bnAb context and HXB2 sites of interest.

HXB2 number	HXB2 aa	Signature region	Test aa	Feature
**22**	L	SP	**L** and **M**	**L** confers neutralisation sensitivity in clade C.
**82**	Q	gp120	**Q** and **R**	VRC34.01 and PGT151 contacts in subtype A and B
**172**	E	V2 loop	**V** and **A**	**V** confers neutralisation sensitivity to VRC26.08. PG16 contact with clade C.
**230**	N	glycosite 230	**D** and **E**	**N** confers neutralisation resistance to PGT127 in clade C. **D** confers neuralisation resitance to 10.1074. **N** confers neutralisation sensitivity to PGT121
**275**	V	D loop	**E** and **K**	**E** confers neutralisation sensitivity to 12A12. K confers neutralisation resistance to VRC13 in subtype C.
**317**	F	V3 loop	**F** and **Y**	Coreceptor binding site inside the V3 loop. Coreceptor-specific (R5/X4) site. Mutations affect neutralisation
**432**	K	CD4 Contact residue	**Q**	VRC27, VRC16 and N60P23 contacts. Amino acid **Q** is confers resistance to 2F5. Amino acid **K** is sensitivity to 2F5.
**476**	R	CD4 Contact residue	**R** and **K**	**R** confers neutralisation sensitivity to CH31, VRC06b, and 8ANC131 in subtype C. **K** confers neutralisation resistance to CH31 VRC06b 8ANC131 **R**476K mutation confers loss of apoptosis induction.
**477**	D	CD4 Contact residue	**D** and **N**	**D**477 is 2411a contacts. N49P7 contact. VRC13 contacts
**784**	L	LLP-2	**L**	**L**784 confers sensitivity 12A12 VRC07 VRC07.523.LS VRC01 in clade C. **L**784**I** mutation confers sensitivity to 3BNC117 in clade C and B. **L**784I mutation Confers resistance to PGDM1400 in SHIV.CAP256SU.375S clade C clone.
**620**	E	c-helix	**D**	**A** confers resistance in subtype C. **E** associated with sensitivity to 2F5 in subtype C
**34**	L	gp120	**L**	Antibody epitope in subtype C
**299**	P	V3 loop	**P**	vFP20.01 functional escape mapping.
**643**	H	Fusion HR-2	**Y**	PGT151 contacts. Significant site of viral escape

## 4 Discussion

The study aimed to detect the unique genetic signatures in the Env of the contemporary HIV-1 T/F viruses among subtypes A1, A1D recombinants and D, which are predominant in East Africa. We identified HIV-1 T/F Env sequences and subtype composition in East Africa, spanning the years 2015 to 2021 in comparison to historical sequences (1986 to 2006). The robust and unique genetic signatures in T/F Envs were analyzed in comparison to historical Envs of the same subtypes. The link between the unique genetic and the Env (gp160) structural characteristics such as sensitivity or resistance to bnAbs, and HXB2 sites of interest was also determined.

We report single variant transmission in acute infections from GHWP, Protocol B and C, and KILIGORIS cohorts. These findings are consistent with previous studies that showed that only one HIV-1 variant traverses the mucosa and establishes a productive infection in 80% of heterosexual cases (Salazar-Gonzalez et al., 2009; Keele et al., 2008) further asserting the existence of a genetic bottleneck during HIV-1 transmission.

In our study of the full length Env gene, subtype A1 was the most frequently transmitted, followed by A1D recombinants and subtype D, consistent with previous reports (Balinda et al., 2022; Bbosa et al., 2019; Kemal et al., 2013; Billings et al., 2017; Gounder et al., 2017). These results suggest that subtype A1 and A1D recombinant Envs possess unique genetic signatures that enhance their transmission and persistence in East Africa. Also, A1D recombinants may benefit from a combination of advantageous genetic features from both parental subtypes.

The comparison between contemporary T/F viruses (2015–2021) and the historical viral population (1986–2006) remains informative for detecting principal genetic signatures associated with viral adaptation at the population level, given that the earliest sequence in the contemporary dataset (2015) is 10 years away from the latest sequence in the historical dataset (2006). This approach provides a meaningful temporal axis to detect unique amino acid signatures, as supported by previous studies (Rademeyer et al., 2016; Cho et al., 2019; Berry et al., 2007; Chaillon et al., 2012).

The present study reports that while the hydrophobic core of the SP domain in subtype A1 T/Fs exhibited a unique LM22 mutation, the historical counterparts retained a conserved L22. Previous studies argue that the SP regulates Env-glycan interactions, which affect antibody binding to the V1V2 configuration, V3 apex, and gp41 epitopes (Lambert and Upadhyay, 2021; Li et al., 2000; Upadhyay et al., 2018; Upadhyay et al., 2020). Thus, the SP is instrumental in escaping host immune responses by altering recognition by antibodies. The emerging M22 signature in contemporary T/F subtype A1 may enhance their transmission fitness in acute infection and persistence in East Africa. The enrichment of the recurrent L22 signature in the SP of historical subtype A1 may sustain long-term infection. Moreover, the robust L22 signature has been associated with neutralisation sensitivity in subtype C viruses (Wang et al., 2011), implying that its enrichment in long-term infections could reflect a trade-off between immune evasion and viral fitness.

While the gp120 domain of T/F subtype A1 exhibited an enrichment of the Q82R mutation, the historical counterparts maintained a conserved Q82 signature site. This may facilitate viral entry into host cells during acute infection by improving receptor binding, while also contributing to viral persistence in long-term infection by influencing T cell immune responses (Santosuosso et al., 2009; Yoon et al., 2010). The robust Q82 signature site is a contact region for VRC34.01 across a geographically diverse panel of HIV strains (Chuang et al., 2019) but mediated escape from PGT15 in a subtype B strain (Dingens et al., 2019). Thus, phenotypic studies are needed to clearly define the role of the emerging Q82R mutation in subtype A1 T/F viruses.

We report a robust V172 signature, highly enriched in the V2 loop of subtype A1 historical viruses, compared to the contemporary T/Fs which exhibited a unique V172A mutation. Its presence may stabilize the Env trimer and maintain its structural integrity on the viral surface in subtype A1 T/Fs while shielding neutralisation-sensitive domains such as the V3 loop and CD4 binding sites in long-term infection (Rao et al., 2013; Rusert et al., 2011). Despite its role in immune evasion, the V172 signature is sensitive to VRC26.08 in group M strains (Roark et al., 2021), revealing potential vulnerabilities that could be targeted for therapeutic intervention. Conversely, the unique A172 signature in subtype A1 T/F Envs and its absence in historical counterparts may alter the local structure and reaction to bnAbs. Future studies should validate the impact of the V172A mutation on the structure and function of V2 loops in subtype A1 T/F viruses.

Previous studies claim that glycosylation sites are critical for immune evasion, enhancing viral persistence in chronic infection (Ho et al., 2008; Zhang et al., 2021), while also stimulating glycan-dependent HIV-neutralizing antibodies that contribute to protective immunity (Zhang et al., 2021). The recurrent D230 signature in historical subtype A1 viruses may support glycosite 230’s role in shielding the virus from immune recognition, allowing persistence. The reduced glycan content in T/F viruses may facilitate infection establishment by enhancing affinity for mucosal surfaces (Nawaz et al., 2011). The elevated E230 site in subtype A1 T/F Envs may contribute to neutralisation resistance in acute infection, thereby enhancing transmission and infectivity. While N230 confers resistance to PGT127 and sensitivity to PGT121 in subtype C Envs, and D230 is linked to resistance to 10.1074 (Bricault et al., 2019), the functional implications of the D230E mutation remain unclear. Future studies must characterize bnAbs that target the E230 signature site in the understudied T/F subtype A1viruses.

In the D loop, the robust E275 signature site was enriched in the historical subtype A1 viruses, whereas the T/F counterparts exhibited the unique K275 signature, which was absent in historical viruses. Mutations in the D loop have been associated with viral resistance to VRC01-like antibodies in subtype B infection (Zhang et al., 2022). Specifically, E275 was linked to sensitivity to 12A12, whereas K275 conferred resistance to VRC13 in subtype C viruses (Bricault et al., 2019). This suggests that the emerging E275K mutation in subtype A1 T/F Envs may enhance viral entry and immune evasion during acute infection, while E275 in historical Envs may contribute to long-term viral persistence.

While the V3 loop of T/F subtype A1 exhibited an enrichment of the unique V317 signature, the historical sequences retained a conserved F317 signature at this site. This contradicts findings from subtype C T/F viruses, where I307 in the V3 loop was less common in acute than chronic infections (Rademeyer et al., 2016). The V317 site in T/F subtype A1 may enhance viral infectivity, influence coreceptor usage and host cell tropism during acute infection. Similarly, the enriched F317 site in the V3 loop of historical sequences may contribute to tropism changes, and sustenance of long-term infection. Mutations in the V3 loop influence neutralisation sensitivity; for example, the F317A mutation in a subtype B strain reduced PGV04 and VRC01 neutralisation but enhanced CD4-IgG and b12 neutralisation (Falkowska et al., 2012). However, the impact of the F317V mutation on bnAbs remains unclear in subtype A1 T/F viruses.

This study highlights that the CD4 contact residues in subtype A1 are under evolutionary pressure in both contemporary T/F and historical sequences. The CD4 contact residues are integral to viral entry. The recurring Q432 signature may aid in sustaining both long-term infection and enhancement of acute subtype T/F A1 transmission. A previous study argues that the R432 residue in the CD4 binding site in subtype C infection confers resistance to 8ANC131 while Q432 and K432 were associated with sensitivity to 2F5 (Bricault et al., 2019). This suggests that the principal Q432 signature in subtype A1 T/Fs could be explored and targeted by therapeutics. Additionally, the emerging R476K mutation in the CD4 contact residue in T/F subtype A1 may enhance viral entry in acute infection while the recurrent R476 in historical counterparts may sustain long-term infection. Structural biology studies claim that R476 is a CD4 contact with a buried surface area (BSA) of 16.5 Å^2^ on BG505 SOSIP.664 (PDB 6CM3) (Stanfield et al., 2020). Functionally, R476 was associated with sensitivity to CH31, VRC06b, and 8ANC131 while K476 confers resistance to CH31 VRC06b 8ANC131 in subtype C (Bricault et al., 2019). A study in subtypes B, C, G, CRF13_cpx, AG/A1, A/E, and A/G recombinants claimed that N425R and R476K mutations were strongly linked to a loss of apoptosis induction (Joshi et al., 2013). This implies that the enriched K476 site in T/F subtype A1 may reduce the fitness of the virus to trigger the death of uninfected CD4 + T cells in acute infection. Phenotypic studies are warranted to understand the function of the R476K mutation in T/F subtype A1 viruses. The great enrichment of the robust D477 signature in the CD477 contact residue of historical sequences may sustain long-term infection. The D477 residue interacts with antibodies such as VRC13, 2411a and N49P7, suggesting that it that could be targeted by therapeutics (Stanfield et al., 2020; Chen et al., 2021; Sajadi et al., 2018). Inversely, the emerging N477 signature in T/F subtype A1 may enhance viral entry by interacting with the CD4 receptor on host cells in acute infection. A previous study reported that D477A mutation significantly decreases b12 binding to less than 50% of wildtype in strain JRCSF (Pantophlet et al., 2003). Therefore, future studies should investigate the impact of the D477N mutation on binding monoclonal antibodies in the context of subtype A T/F viruses. Elucidating the principal amino acid patterns in the CD4bs region across the infection timeline could inform effective therapeutic design.

In the LLP-2 domain, the robust L784 signature was less associated with T/F subtype A1 compared to the historical sequences. The LLPs disrupt membrane permeability, leading to host cell death in subtype D and B infection (Costin et al., 2007). The L784 signature site might enhance the role of LLP-2 domain in perforating the host cell in acute T/F viral infection as well as sustaining long-term subtype A1 infection. Therefore, novel therapies that can target the L784 in LLP-2 could deter formation of viroporin. The recurrent L784 signature site is susceptible to several bnAbs, including 12A12, VRC07, VRC07.523. LS and VRC01 while L784I mutation is sensitive to 3BNC117 in subtype C and B viruses (Bricault et al., 2019). Contrarywise, L784I mutation is associated with resistance to PGDM1400 (Roark et al., 2021). Overall, the evidence suggests that the recurrent L784 signature is a promising target for HIV-1 vaccine development.

Among the A1D recombinants, the robust D620 signature site occurred more in the C-helix of T/F compared to the historical viruses. The D620 in the C-helix within the gp41 domain may enhance membrane fusion during acute T/F infection as well as sustain long-term infection. It is noteworthy that the A620 signature confers resistance to 4E10 while E620 is sensitive to 2F5 in subtype C (Bricault et al., 2019). While these bnAbs target the MPER domain, there is no evidence regarding the impact of D620 site on antibody recognition within the A1D recombinants. Subsequent studies should design bnAbs that target the D620 site in the neglected A1D recombinants. Furthermore, the robust L34 signature site in the gp120 domain was highly selected by A1D recombinant historical Envs compared to the T/F counterparts. The gp120 domain facilitates viral entry into host cells in the acute phase infection by binding to target cell receptors as well as mediating viral persistence by influencing the T cell immune response in the chronics (Santosuosso et al., 2009; Yoon et al., 2010). The recurrent L34 signature may initiate acute infection and sustain long-term infection. The lower frequency of the robust P299 signature in the V3 loop of A1D recombinant T/Fs compared to the historical counterparts may enhance coreceptor usage and host cell tropism throughout infection. A previous study showed that the P299 in the V3 loop of BG505. T332N is a site of viral escape, where P299A have a strong effect and P299H have a moderate effect on immune evasion (Dingens et al., 2019; Zhang et al., 2021). The frequency of the Y643 in the HR2 region occurred less in the A1D recombinant T/Fs compared to historical viruses. The HR2 contributes to transmission and replication by bringing the viral and host cell membranes into proximity. On that note, Y643 could potentially enhance transmission fitness to A1D recombinant T/Fs in the acute phase as well as immune escape in long-term infection. The H643 site in the strain JR-FL interacts with the broadly neutralizing antibody PGT151, which in turn makes it an important target for HIV antibody-based therapeutics (Dingens et al., 2019). Future studies should characterize bnAbs that target the principal Y643 signature site in A1D recombinants.

The low prevalence of subtype D Env sequences in this study limited the detection of informative subtype D specific genetic signatures.

In conclusion, the presence of key genetic signature sites in the SP, gp120, V2, glycosite 230, D loop, V3 loop, CD4 contact residues, and LLP-2 in subtype A1 T/Fs, as well as in the C-helix, gp120, V3 loop, and Fusion HR-2 regions of A1D recombinant T/F Envs, may play a crucial role in the successful establishment of acute and the maintenance of long-term infection. However, phenotypic studies are needed to gain a deeper understanding of how these variations influence viral fitness and immune recognition in the less-studied subtypes A1 and A1D recombinants, which are predominant in East Africa. Also, recognizing that viral persistence also relies on other replication steps such as reverse transcription, integration and assembly, future research should extend the search for unique genetic signatures to other viral proteins mediating these processes in subtypes A1, and A1D recombinants. We acknowledge the limitation of our approach, as comparing contemporary T/F Env sequences from acutely infected individual to unrelated, cross-sectional historical sequences may not account for host specific confounders. While the envelope unique genetic signatures reported in the present study may inform therapeutic interventions, longitudinal cohorts tracking within host evolution from acute to chronic infection are needed to validate these signatures and their functional relevance.

## Data Availability

Publicly available datasets analyzed in this study can be found at the CATNAP webserver http://hiv.lanl.gov/catnap and SFL web alignments https://www.hiv.lanl.gov/content/sequence/NEWALIGN/align.html#filter at LANL HIV db. The accession numbers can be found in the Supplementary Tables S3–S6. Also, original datasets analysed in this study, from acute cohorts (GHWP, protocol N and Kiligoris) were submitted to GenBank under submission ID (2982317).
