# Supplementary material for: Unique genetic signatures in HIV-1 subtype A1 and A1D recombinant envelope glycoprotein distinguish contemporary transmitted/founder viruses from historical strains in East Africa
Source: Front Microbiol. 2025 Aug 4;16:1632581. doi: 10.3389/fmicb.2025.1632581 (PMC12358483; doi:10.3389/fmicb.2025.1632581)
Supplement: Supplementary file 1 [file Data_Sheet_1.docx]

**Supplementary Material**

**Table S 1:** HIV-1 specific SGA - PRC primers

| **Primer ID** | **Primer sequence** | **Primer role** |
| --- | --- | --- |
| b3f1 | ACAGCAGTACAAATGGCAGTATT | 1^st^ round PCR, forward |
| b3f3 | TGGAAAGGTGAAGGGGCAGTAGTAATAC | 2^nd^ round PCR, forward |
| 2.R3.B6R | TGAAGCACTCAAGGCAAGCTTTATTGAGGC | 2^nd^ round PCR, reverse |
| 1.R3.B3R | ACTACTTGAAGCACTCAAGGCAAGCTTTATTG | cDNA synthesis and 1^st^ round PCR, reverse |

**Table S 2:** Characteristics of contemporary HIV-1 T/F Env sequences identified from recent infection cohorts. Mean, median and range number of HIV-1 SGA-derived sequences per participant is 9.56, 9 and 15 respectively.

| **SN** | **Identifier** | **Subtype** | **Country** | **Sampling Year** | **Patient health** | **Cohort** | **Number of SGA sequences** | **GenBank accession** |
| --- | --- | --- | --- | --- | --- | --- | --- | --- |
| 1 | OOG.4529 | A1 | Uganda | 2016 | Acute | GHWP | 19 | PV988136 |
| 2 | OOG.14891 | A1 | Uganda | 2017 | Acute | GHWP | 8 | PV988137 |
| 3 | OOG.03156 | A1 | Uganda | 2016 | Acute | GHWP | 13 | PV988138 |
| 4 | OOG.03467 | A1 | Uganda | 2016 | Acute | GHWP | 8 | PV988139 |
| 5 | OOG.04082 | A1 | Uganda | 2016 | Acute | GHWP | 9 | PV988140 |
| 6 | OOG.15575 | A1 | Uganda | 2017 | Acute | GHWP | 8 | PV988141 |
| 7 | OOG.13862 | A1 | Uganda | 2017 | Acute | GHWP | 7 | PV988142 |
| 8 | OOG.16768 | A1 | Uganda | 2018 | Acute | GHWP | 7 | PV988143 |
| 9 | OOG.12290 | A1 | Uganda | 2017 | Acute | GHWP | 8 | PV988144 |
| 10 | OOG.05321 | A1 | Uganda | 2016 | Acute | GHWP | 13 | PV988145 |
| 11 | OOG.11123 | A1C | Uganda | 2017 | Acute | GHWP | 8 | PV988169 |
| 12 | OOG.03277 | C | Uganda | 2016 | Acute | GHWP | 11 | PV988170 |
| 13 | OOG.03828 | C | Uganda | 2016 | Acute | GHWP | 4 | PV988171 |
| 14 | OOG.14754 | D | Uganda | 2017 | Acute | GHWP | 10 | PV988167 |
| 15 | OOG.10101 | D | Uganda | 2017 | Acute | GHWP | 8 | PV988168 |
| 16 | OOG.11068 | A1D | Uganda | 2017 | Acute | GHWP | 12 | PV988158 |
| 17 | OOG.3176 | A1D | Uganda | 2016 | Acute | GHWP | 11 | PV988159 |
| 18 | OOG.06218 | A1 | Uganda | 2016 | Acute | GHWP | 10 | PV988146 |
| 19 | OOB.12152492 | A1 | Kenya | 2017 | Acute | Protocol N | 11 | PV988147 |
| 20 | OOB.12152566 | A1 | Kenya | 2017 | Acute | Protocol N | 8 | PV988148 |
| 21 | OOB.12152630 | A1 | Kenya | 2016 | Acute | Protocol N | 9 | PV988149 |
| 22 | OOB.12152738 | A1 | Kenya | 2016 | Acute | Protocol N | 11 | PV988150 |
| 23 | OOB.12152787 | A1 | Kenya | 2017 | Acute | Protocol N | 9 | PV988151 |
| 24 | OOB.12152846 | A1 | Kenya | 2017 | Acute | Protocol N | 14 | PV988152 |
| 25 | OOB.12152949 | A1 | Kenya | 2017 | Acute | Protocol N | 12 | PV988153 |
| 26 | OOB.12152628 | A1 | Kenya | 2015 | Acute | Protocol N | 8 | PV988154 |
| 27 | OOB.12152822 | A1 | Kenya | 2017 | Acute | Protocol N | 10 | PV988155 |
| 28 | K060238 | A1 | Uganda | 2021 | Acute | Kiligoris study | 8 | PV988156 |
| 29 | K011703 | A1 | Uganda | 2021 | Acute | Kiligoris study | 11 | PV988157 |
| 30 | 00B.12152913 | A1D | Kenya | 2017 | Acute | Protocol N | 7 | PV988160 |
| 31 | 00B.12152796 | A1D | Kenya | 2017 | Acute | Protocol N | 9 | PV988161 |
| 32 | K020157 | A1D | Uganda | 2021 | Acute | Kiligoris | 8 | PV988162 |
| 33 | K020001 | A1D | Uganda | 2021 | Acute | Kiligoris | 8 | PV988163 |
| 34 | K030189 | A1D | Uganda | 2021 | Acute | Kiligoris | 12 | PV988164 |
| 35 | K040020 | A1D | Uganda | 2021 | Acute | Kiligoris | 8 | PV988165 |
| 36 | K030784 | A1D | Uganda | 2021 | Acute | Kiligoris | 7 | PV988166 |

**Table S 3:** Accessions/sequence names of publicly available contemporary HIV-1 T/F sequences from East Africa (2015 – 2021) in the LANL HIV database (CATNAP <https://www.hiv.lanl.gov/components/sequence/HIV/neutralization/download_db.comp> and SFL alignments <https://www.hiv.lanl.gov/content/sequence/NEWALIGN/align.html> ).

| MZ642260 |
| --- |
| MZ642261 |
| MZ642262 |
| MZ642265 |
| MZ642266 |
| MZ642267 |
| MZ642269 |
| MZ642278 |

**Table S 4**: Accessions/sequences names of publicly available HIV-1 historical subtype A1 sequences from East Africa (2006 – 1986) in the LANL HIV database (CATNAP and SFL alignments)

| DQ208456 | AF407151 | AY736813 | AY669703 | KT022363 |
| --- | --- | --- | --- | --- |
| DQ208458 | AF407154 | FJ866117 | EU855131 | KT022364 |
| A1.KE.1993.BG505_W6M_C2_N301A | AF407155 | EU852958 | U08794 | KT022365 |
| A1.KE.1993.BG505_W6M_C2_N611Q | AF407156 | FJ866113 | AY669697 | KT022367 |
| A1.KE.1993.BG505_W6M_C2_T332N | AF407157 | FJ866115 | HM215347 | KT022368 |
| AF407160 | AF407158 | FJ866116 | HM215299 | KT022369 |
| AF407161 | AF407159 | FJ866119 | HM215348 | KT022370 |
| AF407162 | FJ866111 | FJ866120 | HM215298 | KT022372 |
| AF484493 | AY736809 | FJ866121 | HM215306 | KT022373 |
| AF407152 | AY736810 | FJ866122 | HM215255 | KT022374 |
| AF407153 | AF457066 | FJ866118 | HM215258 | KT022375 |
| A1.RW.1992.92RW020_2_T303A | HM215256 | HM215257 | HM215334 | KT022376 |
| AY669701 | U09127 | AY736812 | AF457055 | KT022377 |
| A1.UG.1992.UG037_8_K651N | AY736815 | AY736816 | AF457053 | KT022380 |
| AF457070 | AF457080 | AF457081 | AF457084 | KT022381 |
| AF457086 | MW383940 | KT022360 | KT022361 | KT022382 |
| KT022383 | FJ623487 | FJ623481 | FJ623475 | FJ623476 |
| FJ623478 | FJ623482 | FJ623477 | FJ623486 | FJ623479 |
| AY322184 | AF539405 | L22943 | KX168075 | AF004885 |
| KT008652 | FJ866112 | AY322190 | AY322193 | FJ396018 |
| AY736811 | AY736814 | AF457075 | MT942764 | MT942776 |
| KX983643 | MT942721 | MT942795 | AB253421 | AB287377 |
| AB287379 | AY713406 | HQ659604 | HQ659597 | HQ697994 |
| HQ698029 | AY253305 | AY253314 | KX907352 | KX907348 |
| KX907336 | KX907341 | KX907347 | KX907364 | KX907412 |
| KX907414 | KX907423 | KX907431 | AF361872 | AF361873 |
| L22957 | AB253429 | AY494973 | EU853062 |  |
| EU852954 | AF484507 | AF484509 | EU853030 |  |

**Table S 5:** Accessions/sequences names of publicly available HIV-1 Historical A1D recombinants sequences from East Africa (2006 – 1986) in the LANL HIV database (CATNAP and SFL alignments)

| KT022404 | KT022410 | KX907355 | A1D.KE.x.MS208_A3 | DQ208497 |
| --- | --- | --- | --- | --- |
| HM027864 | KT022385 | AF484492 | AF442565 | KT022416 |
| HM027827 | AF484488 | KT022388 | KT022387 | KT022417 |
| KT022400 | HM215280 | KT022401 | AF407148 | FJ866128 |
| KX907351 | AF457056 | KT022409 | AF407149 | AY945737 |
| MN791535 | AF442566 | AF484503 | DQ208493 | U36866 |
| KT022411 | HM215301 | KJ579955 | DQ208499 | KT022394 |
| AF484479 | KX907376 | KX364401 | DQ208492 | AF484482 |
| KT022399 | KT022390 | KT022397 | EF575459 | HQ698035 |
| L22942 | DQ208480 | FJ866127 | AF457058 | U36883 |
| DQ208425 | DQ208428 | DQ208431 | DQ208433 | AY352657 |
| AF484510 |  |  |  |  |

**Table S 6:** Accessions/sequences names of publicly available HIV-1 historical subtype D sequences from East Africa (2006 – 1986) in the LANL HIV database (CATNAP and SFL alignments)

| KF986074 | EF575429 | JQ715411 | AY494966 | JX512899 |
| --- | --- | --- | --- | --- |
| KF986041 | U36886 | HM215283 | AY669751 | JX236668 |
| U36871 | FJ866137 | AY669758 | AY736829 | HM215270 |
| EF575364 | FJ866134 | AY623599 | HM215356 |  |
| EF575370 | FJ866138 | U27399 | HM215357 |  |
| EF575388 | FJ866139 | U43386 | HM215358 |  |
| EF575400 | FJ866140 | HM215353 | AY736832 |  |
| EF575417 | FJ866141 | AY669750 | JQ361079 |  |

**Table S 7:** Contemporary T/F subtype A1 amino acid frequency by position

cutoff: 95%

|  | **Percentage and raw  count of non-gap** | **Non-gap/total (percentage)** | **Gap/total (percentage)** |
| --- | --- | --- | --- |
| 22 | L: 80.00% (24)   F: 10.00% (3)   M: 6.67% (2)   W: 3.33% (1) | 30/30 (100.00%) | 0/30 (0.00%) |
| 82 | Q: 86.67% (26)   R: 13.33% (4) | 30/30 (100.00%) | 0/30 (0.00%) |
| 172 | V: 70.00% (21)   E: 10.00% (3)   A: 6.67% (2)   I: 6.67% (2)    T: 3.33% (1)   M: 3.33% (1) | 30/30 (100.00%) | 0/30 (0.00%) |
| 230 | D: 63.33% (19)   E: 23.33% (7)   O: 6.67% (2)   M: 3.33% (1)    N: 3.33% (1) | 30/30 (100.00%) | 0/30 (0.00%) |
| 275 | E: 80.00% (24)   K: 13.33% (4)   A: 3.33% (1)   Q: 3.33% (1) | 30/30 (100.00%) | 0/30 (0.00%) |
| 317 | F: 86.67% (26)   Y: 6.67% (2)   L: 3.33% (1)   I: 3.33% (1) | 30/30 (100.00%) | 0/30 (0.00%) |
| 432 | Q: 70.00% (21)   K: 10.00% (3)   R: 10.00% (3)   L: 3.33% (1)    T: 3.33% (1)   E: 3.33% (1) | 30/30 (100.00%) | 0/30 (0.00%) |
| 476 | R: 76.67% (23)   K: 23.33% (7) | 30/30 (100.00%) | 0/30 (0.00%) |
| 477 | D: 90.00% (27)   N: 10.00% (3) | 30/30 (100.00%) | 0/30 (0.00%) |
| 784 | L: 86.21% (25)   I: 6.90% (2)   R: 3.45% (1)   T: 3.45% (1) | 29/30 (96.67%) | 1/30 (3.33%) |

**Table S 8:** Historical Env Subtype A1 amino acid frequency by position

cutoff: 95%

|  | **Percentage and raw  count of non-gap** | **Non-gap/total (percentage)** | **Gap/total (percentage)** |
| --- | --- | --- | --- |
| 22 | L: 99.28% (137)   F: 0.72% (1) | 138/138 (100.00%) | 0/138 (0.00%) |
| 82 | Q: 98.55% (136)   R: 1.45% (2) | 138/138 (100.00%) | 0/138 (0.00%) |
| 172 | V: 92.03% (127)   E: 5.07% (7)   other: 2.90% (4) | 138/138 (100.00%) | 0/138 (0.00%) |
| 230 | D: 86.96% (120)   E: 9.42% (13)   O: 3.62% (5) | 138/138 (100.00%) | 0/138 (0.00%) |
| 275 | E: 96.38% (133)   other: 3.62% (5) | 138/138 (100.00%) | 0/138 (0.00%) |
| 317 | F: 98.55% (136)   other: 1.45% (2) | 138/138 (100.00%) | 0/138 (0.00%) |
| 432 | Q: 88.41% (122)   R: 7.97% (11)   K: 3.62% (5) | 138/138 (100.00%) | 0/138 (0.00%) |
| 476 | R: 95.65% (132)   K: 4.35% (6) | 138/138 (100.00%) | 0/138 (0.00%) |
| 477 | D: 100.00% (138) | 138/138 (100.00%) | 0/138 (0.00%) |
| 784 | L: 98.53% (134)   other: 1.47% (2) | 136/138 (98.55%) | 2/138 (1.45%) |

**Table S 9:** Contemporary T/F A1D recombinant amino acid frequency by position

Cutoff:95%

|  | **Percentage and raw  count of non-gap** | **Non-gap/total (percentage)** | **Gap/total (percentage)** |
| --- | --- | --- | --- |
| 34 | L: 55.56% (5)   W: 22.22% (2)   S: 11.11% (1)   G: 11.11% (1) | 9/9 (100.00%) | 0/9 (0.00%) |
| 299 | P: 77.78% (7)   V: 11.11% (1)   L: 11.11% (1) | 9/9 (100.00%) | 0/9 (0.00%) |
| 620 | D: 66.67% (6)   E: 33.33% (3) | 9/9 (100.00%) | 0/9 (0.00%) |
| 643 | Y: 77.78% (7)   F: 22.22% (2) | 9/9 (100.00%) | 0/9 (0.00%) |

**Table S 10:** Historical A1D recombinant amino acid frequency by position

Cutoff:95%

|  | **Percentage and raw  count of non-gap** | **Non-gap/total (percentage)** | **Gap/total (percentage)** |
| --- | --- | --- | --- |
| 34 | L: 96.36% (53)   other: 3.64% (2) | 55/56 (98.21%) | 1/56 (1.79%) |
| 299 | P: 100.00% (56) | 56/56 (100.00%) | 0/56 (0.00%) |
| 620 | E: 25.00% (14)   D: 23.21% (13)   S: 23.21% (13)   N: 12.50% (7)    Q: 3.57% (2)   H: 3.57% (2)   K: 3.57% (2)   T: 1.79% (1)    other: 3.57% (2) | 56/56 (100.00%) | 0/56 (0.00%) |
| 643 | Y: 100.00% (56) | 56/56 (100.00%) | 0/56 (0.00%) |
